# Supplementary material for: Impact of body composition on patient prognosis after SARS-Cov-2 infection
Source: PLoS One. 2023 Jul 28;18(7):e0289206. doi: 10.1371/journal.pone.0289206 (PMC10381082; doi:10.1371/journal.pone.0289206)
Supplement: S1 Table — (DOCX) [file pone.0289206.s001.docx]

**S1 Table. Comparison of IMAC between survivors and non-survivors in each obesity grade in severe patients**

|  | Patient number | | IMAC | |  |
| --- | --- | --- | --- | --- | --- |
|  | Survivors  (n=35) | Non-survivors  (n=13) | Survivors  (n=35) | Non-survivors  (n=13) | p-values |
| Obesity grade 0 | 6 | 2 | -0.20 | -0.30 | 0.314 |
| Obesity grade 1 | 14 | 7 | -0.35 | -0.35 | 0.709 |
| Obesity grade 2-4 | 15 | 4 | -0.27 | -0.30 | 0.515 |

IMAC, intra-muscular adipose tissue content.

Data are expressed as medians and ranges. The Mann-Whitney U test was used for comparison.
