# Supplementary material for: Impact of body composition on patient prognosis after SARS-Cov-2 infection
Source: PLoS One. 2023 Jul 28;18(7):e0289206. doi: 10.1371/journal.pone.0289206 (PMC10381082; doi:10.1371/journal.pone.0289206)
Supplement: Attachment — Submitted filename: Resonse to Reviewers.docx [file pone.0289206.s003.docx]

Professor Jun Mori
Academic Editor
PLOS ONE

PONE-D-23-01341R1

Impact of body composition on patient prognosis after SARS-Cov-2 infection

Dear Prof. Jun Mori

We appreciate your positive response and the opportunity to address the reviewer’s and editor’s comments regarding our submitted manuscript (PONE-D-23-01341R1). We would also like to take this opportunity to thank the reviewer for the time and insightful suggestions. We have modified our manuscript according to the reviewer’s suggestions. As requested, a point by point response to the issue raised by the reviewer now follows:

**Reviewer #1: I must refer to the discussion**.
**The author must precisely read the references you used, especially Ref.31 written by Bay ML. He precisely described that anti-inflammatory effects are induced by the muscle through physical exercise.
The author inserted new discussion, using the review article (Ref.31). That article shows many kinds of myokine induced by physical exercise. Unfortunately, author’s description in the discussion was equivocal.
I understand that the muscle-derived (md) IL-6 increases by exercise, and mdIL6 suppresses TNF-alfa production, through that review article. Certainly, mdIL6 is anti-inflammatory cytokine. Exercise may be protective to inflammation, or COVID19 pneumonia. This context that the author wrote would be correct.
However, I wonder if intramuscular hyperadiposity, which means high IMAC in the muscle, could lead to inflammatory excitement in the lung or immune system in patients with COVID19 pneumonia. This discussion matters.
Moreover, I would like to know whether intramuscular adipocyte specifically secrets inflammatory cytokines and adipokines. Or I would like to know whether there could be crosstalk between the muscle and the adipocytes in muscle in immune response. Such discussion is of importance, and should be added, and be written concretely. Discussion should be more profound and meaningful.**

We greatly appreciate this comment. We agree our discussion about myokines is equivocal, therefore, we focused on IL-6 in the revised version, for it is one of the most important and well-studied inflammatory myokine. It is reported that increased level of IL-6 is produced by the muscle tissues with fatty regeneration (Addison O, et al.). As the reviewer pointed out, muscle-derived IL-6 has some anti-inflammatory effect. However, IL-6 has two-way signal transductions by the differed receptors. One signaling (classic signaling) induces generative and protective responses to small subset of cells (hepatocytes and leucocytes), the other signaling (trans-signaling) induces proinflammatory responses to all cells in the body. Moreover, it is reported that IL-6 plays a crucial role for the pathogenesis of cytokine release syndrome which leads disease severity of COVID-19. In muscle cells, though IL-6 has some beneficial effects for muscle growth, myogenesis or energy metabolism, it also has negative effects such as inducing muscle atrophy or wasting that could affect COVID-19 prognosis.

We added these things concisely and modified discussion section to make it less equivocal and more profound. We also replaced Ref 31 to another references (Ref. 31, 32, 33) which more adequately support our description.

Once again, we would like to thank you and the reviewer for the positive suggestion. We believe our manuscript has been further improved as a result of incorporation of the reviewer’s suggestion and hope you will now find it suitable for publication in *PLOS ONE*.

Norihiko Yamamoto M.D., Ph.D.

Department of General Medicine

Mie University Hospital, 2-174 Edobashi, Tsu, Mie 514-8507, Japan

Tel: +81-59-231-5290

Fax: +81-59-232-5289
