# Supplementary material for: Impact of body composition on patient prognosis after SARS-Cov-2 infection
Source: PLoS One. 2023 Jul 28;18(7):e0289206. doi: 10.1371/journal.pone.0289206 (PMC10381082; doi:10.1371/journal.pone.0289206)
Supplement: Attachment — Submitted filename: Response to Reviewer (1).docx [file pone.0289206.s004.docx]

Professor Jun Mori
Academic Editor
PLOS ONE

PONE-D-23-01341R2

Impact of body composition on patient prognosis after SARS-Cov-2 infection

Dear Prof. Jun Mori

We appreciate your positive response and the opportunity to address the reviewer’s and editor’s comments regarding our submitted manuscript (PONE-D-23-01341R2). We would also like to take this opportunity to thank the reviewer for the time and insightful suggestions. We have modified our manuscript according to the reviewer’s suggestions. As requested, a point by point response to the issue raised by the reviewer now follows:

**Reviewer #1:**

**Obesity can also predict the severity of COVID19 pneumonia in Japan as well as in the United States and European countries. In Japan, the population of severe obesity is fewer than in the United States and European countries. The current study suggests that IMAC might be predictive for the severity of COVID19 pneumonia in non-obese Japanese patients. In that context, to discover a predictor on the severity of COVID19 pneumonia is greatly meaningful in Japan. The finding that the author discovered on IMAC in this study is interesting to me.
However, IMAC does not look different in the Japanese obese patients with COVID19 pneumonia. So, IMAC may not be useful to predict the severity of COVID19 pneumonia as well as mortality in Japan.
The author should modify the conclusion in the abstract in order to avoid misunderstanding.**

We agree this comment. Our description in the Abstract may cause misunderstanding. As the reviewer stated, our data showed IMAC was different between severe and non-severe COVID-19 cases in non-obese patients. And it does not predict disease course in obese patients or disease mortality in any obesity grade. We modified the conclusion in the Abstract to make these points clearer according to the reviewer’s suggestion.

Once again, we would like to thank you and the reviewer for the positive suggestion. We believe our manuscript has been further improved as a result of incorporation of the reviewer’s suggestion and hope you find it meet *PLOS ONE’s* publication criteria now.

Norihiko Yamamoto M.D., Ph.D.

Department of General Medicine

Mie University Hospital, 2-174 Edobashi, Tsu, Mie 514-8507, Japan

Tel: +81-59-231-5290

Fax: +81-59-232-5289
